# Haplotype diversity of 17 Y-STR in the Iranian population

**DOI:** 10.1186/s12864-024-10217-1

**Published:** 2024-04-02

**Authors:** Mohammad Reza Eskandarion, Arash Alipour Tabrizi, Reza Shirkoohi, Reza Raoofian, Masume Naji, Reza Pazhoomand, Hooman Salari, Bahram Samadirad, Alireza Sabouri, Mostafa Montazer Zohour, Hadi Namazi, Pegah Farhadi, Zohre Baratieh, Minoo Sayyari, Maliheh Dadgarmoghaddam, Esmat Safdarian, Afrooz Nikbakht, Farnaz Golshan, Fatemeh Baybordi, Elham Madhaji, Shadi ShohodiFar, Mohsen Tabasi, Ramezan Mohebbi

**Affiliations:** 1 Legal Medicine Research Center, Legal Medicine Organization, Tehran, Iran; 2 Cancer Research Institute, Imam Khomeini Hospital Complex, Tehran University of Medical Sciences, Tehran, Iran; 3 Plant Genetics and Production, Razi University, Kermanshah, Iran; 4 Department of Community Medicine, School of Medicine, Mashhad University of Medical Science, Mashhad, Iran; 5 Department of Molecular Biology, Pasteur Institute of Iran, Pasteur Ave, Tehran, Iran

**Keywords:** Forensic Genetics, Population Genetic, Y-STR, Iranian Population, Y chromosome haplotype

## Abstract

The current study aimed to evaluate Y chromosome haplotypes obtained from 1353 unrelated Iranian males using the AmpFlSTRTM YfilerTM kit; 1353 out of the 1353 identified haplotypes were unique. The haplotype diversity (HD) and discriminating capacity (DC) values were 1.00000 and 0.997, respectively. Analysis of genetic distance was performed using molecular variance (AMOVA) and multidimensional scaling plots (MDS), revealing a statistically significant difference between the study population and previous data reported for other Iranian populations and other neighboring countries. The present findings are likely to be useful for forensic casework analyses and kinship investigations.

**Supplementary Information:**

The online version contains supplementary material available at 10.1186/s12864-024-10217-1.

## Introduction

There is a 0.3% variation in human DNA among different individuals [1]. The use of this partial difference, which is the criterion for the unity of every human being, makes it possible to distinguish the identity of an individual from another [1]. The genetic identity dataset or genetic profile of a person consists of a combination of alleles obtained from several genetic locations. Therefore, the diagnosis of genetic identity or genetic fingerprinting refers to the process by which the genotype is found in specific regions. It is obtained, recorded, and interpreted from the DNA molecules of an individual [2]. The analysis of variation in the number of these repetitions, called polymorphism, among populations or individuals of a population provides useful markers for population genetic studies, anthropology, and forensic studies [3]. Y chromosomal short tandem repeat (Y-STR) markers can be used as valuable tools in forensic science [4]. Roewer et al. (1992) provided a description of the first polymorphic Y-chromosome marker, Y-27 H39, which is now better known as the STR locus DYS19. Y-STR shows a unique haplotype because the parts carrying Y-STR in the Y chromosome have no homologues on the X chromosome. Because they are passed from father to son, determining the chromosomal Y-STR, tracing, and following the paternal lineage are feasible. Y-STR sequences are used to identify male individuals, but they are better used for population genetics because Y-chromosome haplotype diversity is a useful tool for studying human migration and evolutionary patterns and is also commonly used in genealogical studies [5]. Other regions of the Y chromosome, except for the pseudo-autosomal region, do not contribute to recombination with the X chromosome during meiosis; therefore, this Y chromosome region has less haplotype diversity than autosomal chromosomes. To increase the power of differentiation, more specific Y-STR markers with more specific use should respond to the intra- and extra-population diversity of these markers [6].

Iran covers an area of 1,648,195 sq. km and is the second-largest country in the Middle East, followed by Saudi Arabia. According to the latest census data, Iran’s population was 84,181,200 on January 9, 2021, composed of various ethnic, linguistic, dialect, and religious minorities who have been living in the country for a long time due to a high rate of inbreeding marriages that vary within each population [7–9]. Iran shares its northwest borders with Armenia and Azerbaijan, northeast with Turkmenistan, east with Afghanistan, southeast with Pakistan, and on its western borders with Turkey and Iraq. It also shares borders through the Caspian Sea in the north and the Persian Gulf and Gulf of Oman in the south [10].

Iran was one of the largest empires in history since the 6th century B.C. It took multitudinous forms of migration and invasion by countries from near and far, indicating the importance of studying Iran’s population more than ever [8, 9].

Few studies have been conducted on Iranian Y-STR haplotypes in different geographical regions of the country [11–15]; however, the available data seem to be insufficient. Accordingly, there is a need to perform studies on Iranians based on Y-STR markers.

## Materials and methods

### Sampling and DNA extraction

Haplotype analysis and genetic testing of the 17 Y-STR loci ( DYS19, DYS385a, DYS385b, DYS389I, DYS389II, DYS390, DYS391, DYS392, DYS393, DYS437, DYS438, DYS439, DYS448, DYS456, DYS458, DYS635, and YGATA H4) were performed on a large sample representative of nearly the entire Iranian population. Table 1 presents characteristics of the 17 Y-STR loci.

**Table 1:** The characteristics of Y-STRP markers used in this study

	Y-STR	Allele range
1	DYS456	13–18
2	DYS389 I	9–17
3	DYS390	17–28
4	DYS389 II	23–34
5	DYS458	14–20
6	DYS19 / DYS394	10–19
7	DYS385a/b	7–25
8	DYS393	8–16
9	DYS391	6–14
10	DYS439	8–15
11	DYS635	20–26
12	DYS392	7–18
13	Y GATA H4	8–13
14	DYS437	13–17
15	DYS438	8–13
16	DYS448	17–27
17	DYS456	13–18

**Fig. 1:**
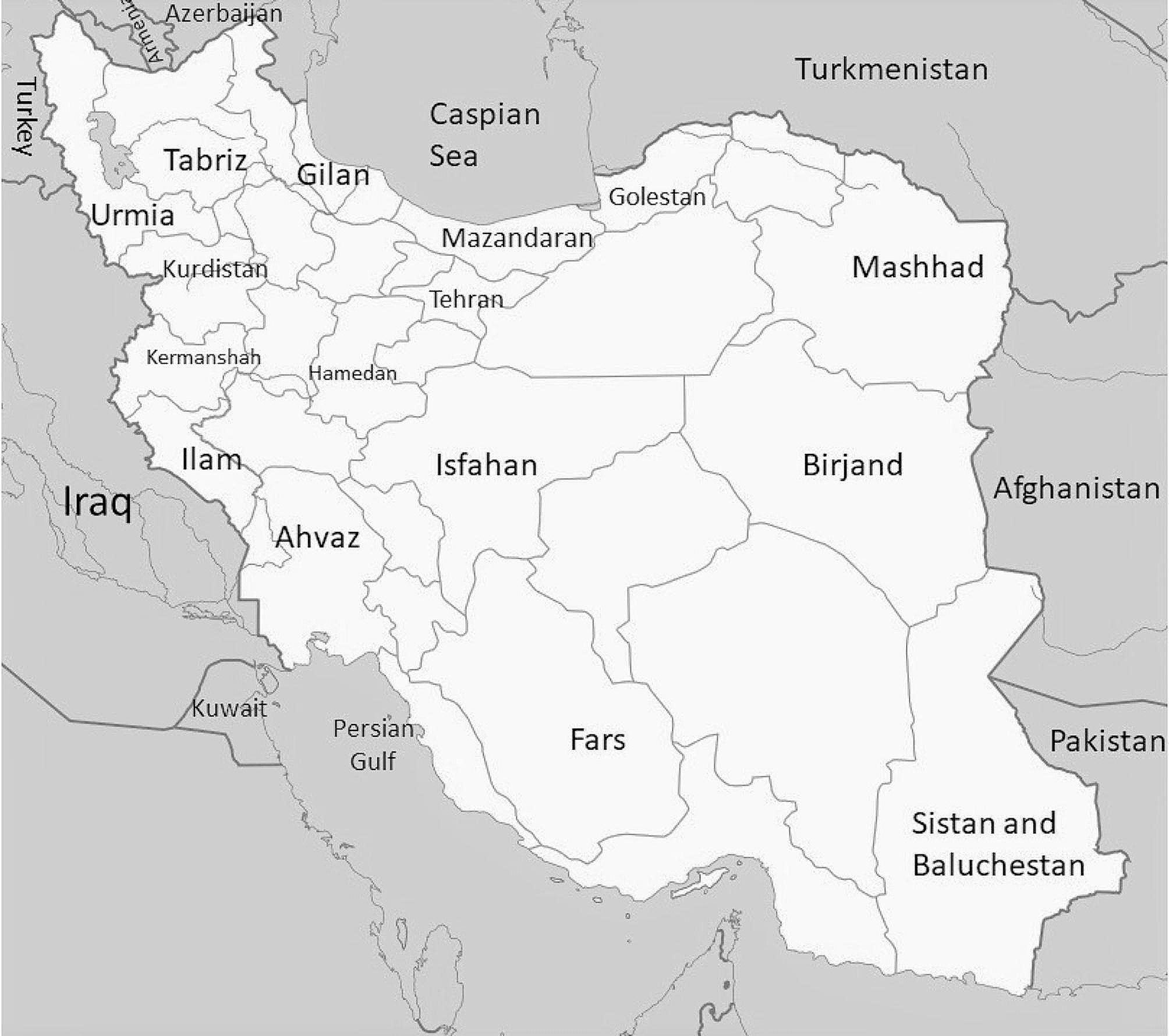
Map of different geographical regions of Iran and the neighboring countries

The study population consisted of 1353 male individuals from different provinces of Iran with different socio-geographical regions, including Tehran (Farsi, *n* = 236) and Isfahan (Farsi, *n* = 153) in the capital and center of Iran; Golestan (Tabari-Farsi, *n* = 82), Mazandaran (Mazani, *n* = 102), and Gilan (Gilak, *n* = 77) in the north; Tabriz (Azari, *n* = 41) and Urmia (Azari, *n* = 53) in the northwest; Kermanshah (Kurdi, *n* = 75), Kurdistan (Kurdi, *n* = 50), Ilam (Luri, *n* = 55), and Hamedan (Farsi, *n* = 34) in the west; Fars (Farsi, *n* = 105) and Khuzestan (Arabic and Farsi, *n* = 34) Moreover, 256 male Iranian individuals were from Khorasan Razavi (Farsi, *n* = 127), South Khorasan (Farsi, *n* = 27), and Sistan and Baluchestan (Sistani-Farsi, *n* = 102), lying in the east and southeast, and the population results were previously published by us [11] as a pilot study and letter to the editor. Hence, the results and samples were employed to comprehensively analyze the Iranian population (Fig. 1).

The study was conducted in 16 forensic genetic laboratories throughout Iran to perform the genetic tests. None of the sample donors were related to each other in at least three generations of blood-related families. Sampling was performed from during 2019–2020.

Each participant read and signed a written informed consent form following the general principles outlined in the Declaration of Helsinki [16]. The Ethics Committee of the Legal Medicine Organization of Iran approved the study protocol (IR.LMO.REC.1398.022).

### DNA extraction and genotyping

Freshly drawn blood samples were taken from the subjects using FTA® classic cards developed by Whatman FTA® technology. For DNA extraction, 1.2 mm punches were used, and DNA was extracted according to the manufacturer’s instructions. The 17 Y-STR loci (AmpFlSTRTM YfilerTM kit, Life Technologies) were simultaneously increased using the Veriti ® Thermal cycler (Applied Biosystems) according to the manufacturer’s protocols. Capillary electrophoresis on automated ABI 3500 and 3130 Genetic Analyzers (Applied Biosystems, Foster City, CA, USA) was used to carry out the electrophoresis of the amplified PCR products. Haplotype analysis was conducted using Genemapper ID version 3.2 and Genemapper IDX vesrsion 1.4 (Applied Biosystems, Foster City, CA, USA). Both DNA typing and nomenclature assignments were performed in accordance with ISFG recommendations [17].

### Data Analysis

Haplotype diversity was computed using Nei’s Eq. [18] and HapYDive [19, 20]. Discriminatory capacity was computed by dividing the number of haplotypes by the total study population [21]. The haplotype match probability (HMP) was computed using the following formula: HMP = 1–HD. The genetic distance among the study population within Iran and between Iran and neighboring countries was calculated using the F_ST_ value from the YHRD [22–24].

## Results

### Alleles Diversity

The data obtained from 17 Y-STR haplotypes were made accessible via the YHRD (Y Chromosome Haplotype Reference Database (http://www.yhrd.org), consisting of 16 distinct accession numbers: YA004580, YA004228, YA004259, YA004258, YA004257, YA004586, YA004587, YA004584, YA004585, YA004583, YA004582, YA004229, and YA004581 for Tehran, Isfahan, Golestan, Mazandaran, Gilan, Mashhad, Birjand, Sistan and Baluchestan, Tabriz, Urumieh, Kermanshah, Kurdistan, Ilam, Hamedan, Shiraz, and Ahvaz, respectively. 1353 of the 1353 haplotypes identified were unique (Supplementary Table S1). The haplotype diversity and discriminating capacity values were 1.00000 and 0.997, respectively. The HMP for the total Iranian population was estimated at 0.0000. The value of genetic diversity ranged from 0.340 (DYS391 in Tabriz) to 0.873 (DYS385a in Mazandaran), which was calculated using the F_ST_ value from the YHRD database [22–24].

**Fig. 2:**
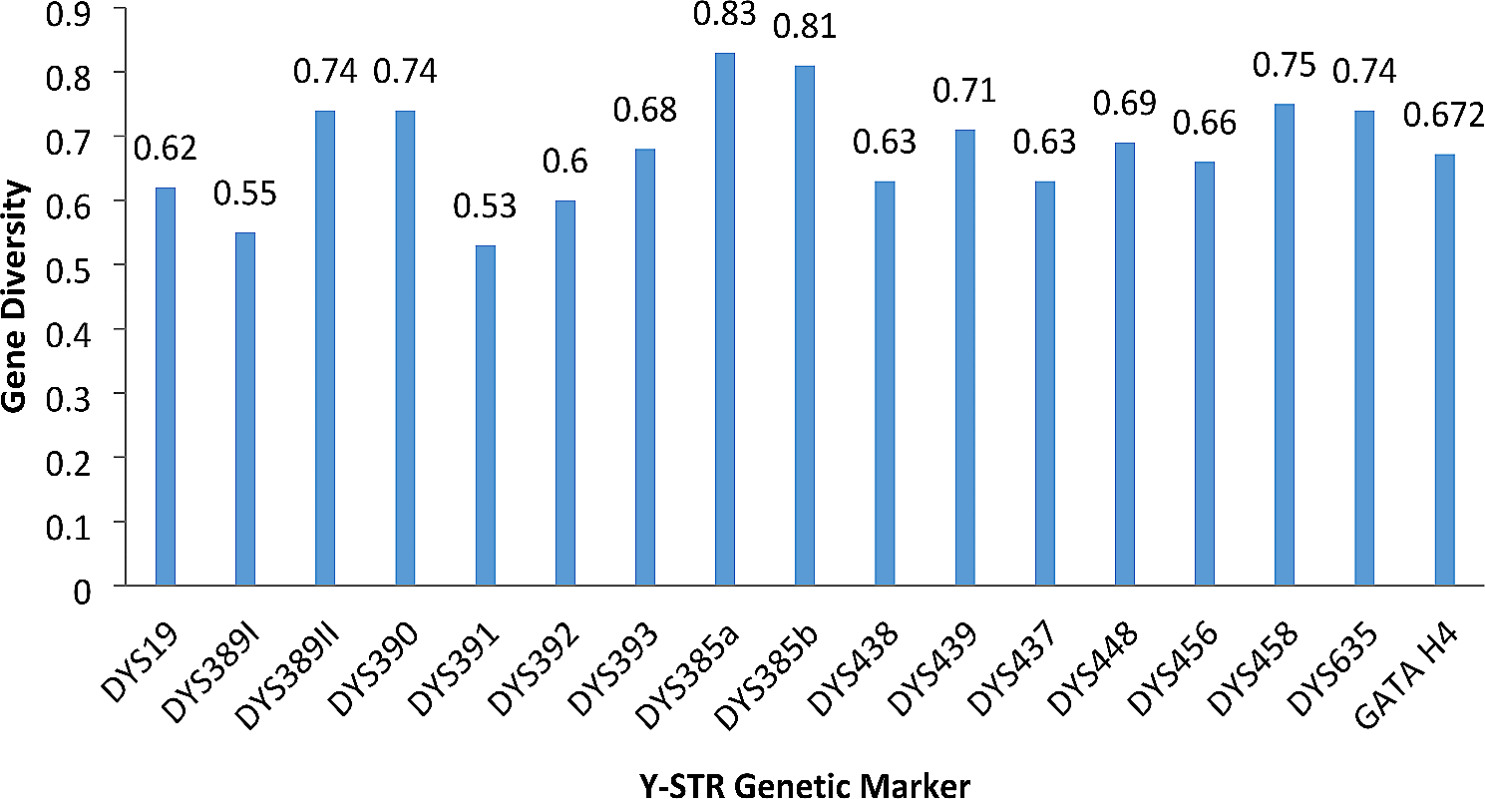
Gene diversity in the study population

The highest polymorphic content in the Iranian population was observed in DYS385a, whereas the lowest polymorphic STRP marker was found in DYS391. The mean gene diversity of the Iranian population was 0.685 (Supplementary Table S2, Fig. 2).

### Population comparison

Among the 17 markers, DYS385 produced double fragments (a and b) because the primers used for this marker showed 100% homology in more than one location of Y chromosome sequences. The results obtained from the current AMOVA analysis and previous studies (28 different geographical regions of Iran) are presented in Supplementary Tables 3, and the AMOVA analyses performed on different regions of Iran and neighboring countries are provided in Supplementary Table 4.

**Fig. 3:**
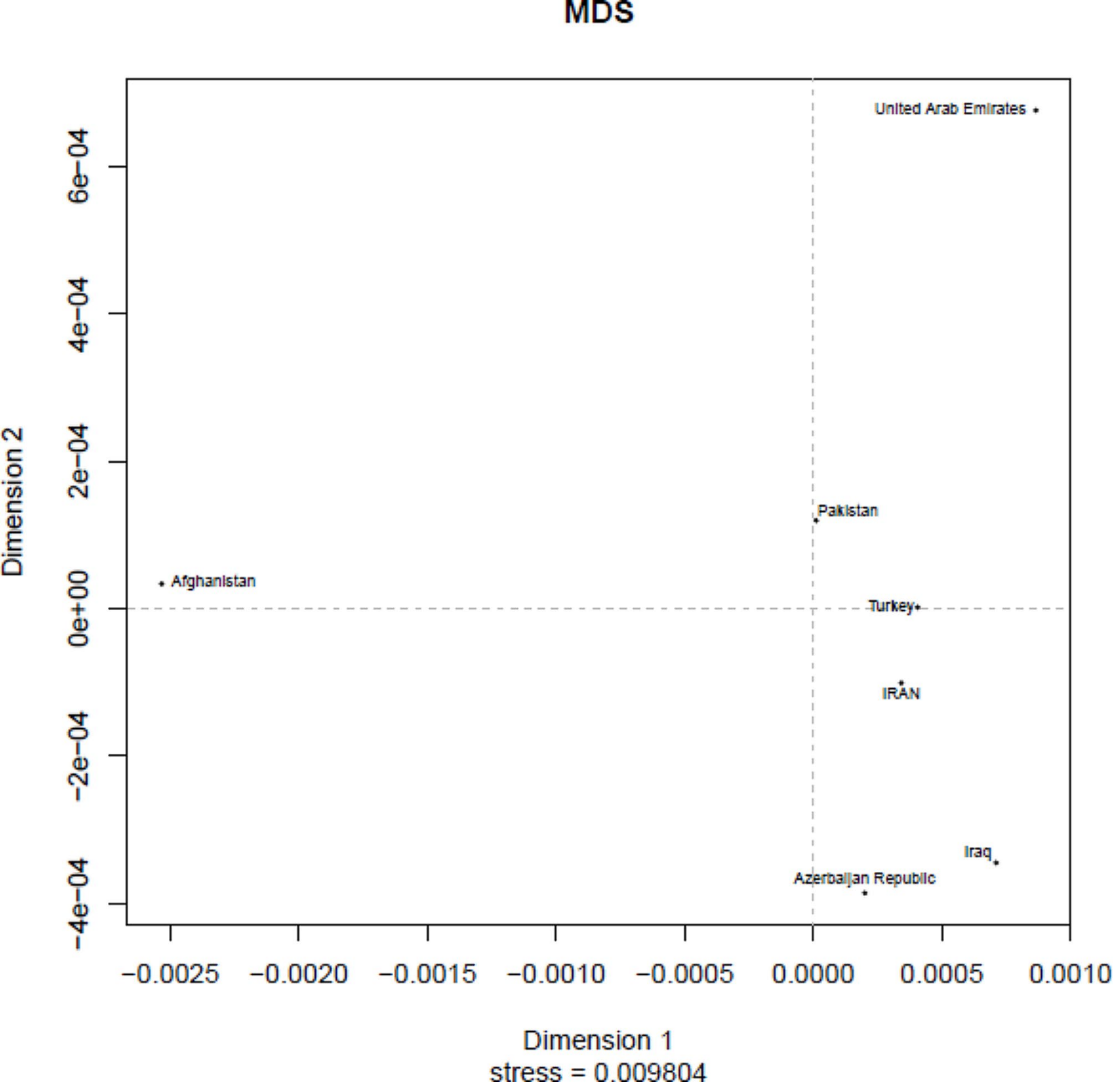
Multidimensional scaling (MDS) plot based on pairwise FST values between the study population and neighboring populations in the YHRD database

Table 2 also shows a comparison of pairwise genetic distances (F_ST_) between Iranians and neighboring populations, including Afghanistan, Pakistan, Turkey, Azerbaijan Republic, Iraq, the United Arab Emirates, Bahrain, Egypt, Kazakhstan, Kuwait, Lebanon, Mongolia, Russian Federation, Qatar, and Yemen in the YHRD database. These F_ST_ values were provided by the clustering patterns found in the multidimensional scaling (MDS) plot according to the F_ST_ values (Fig. 3).

**Table 2:** A comparison of pair wise genetic distance (FST) between Iranians and neighboring populations in the YHRD database

Population	IRAN	Bahrain	Egypt	Kazakhstan	Kuwait	Lebanon	Mongolia	Qatar	Russian Federation	Yemen
IRAN	-	0	0	0	0	0	0	0	0	0.0001
Bahrain	0.0014	-	0	0	0	0	0	0	0	0.0037
Egypt	0.0008	0.0022	-	0	0	0	0	0	0	0.0055
Kazakhstan	0.0037	0.0051	0.0046	-	0	0	0	0	0	0.001
Kuwait	0.0018	0.0028	0.0026	0.0055	-	0	0	0	0	0.0011
Lebanon	0.0014	0.0028	0.0022	0.0051	0.0032	-	0	0	0	0.0054
Mongolia	0.0031	0.0046	0.004	0.0051	0.0049	0.0046	-	0	0	0.002
Qatar	0.0015	0.0026	0.0024	0.0053	0.0032	0.003	0.0047	-	0	0.0031
Russian Federation	0.0003	0.0017	0.0011	0.0039	0.0021	0.0017	0.0032	0.0019	-	0.0079
Yemen	0.0007	0.0021	0.0015	0.0045	0.0025	0.0021	0.0039	0.0021	0.001	-

Population	IRAN	Afghanistan	Azerbaijan Republic	Iraq	Pakistan	Turkey	United Arab Emirates
IRAN	-	0	0	0	0	0	0
Afghanistan	0.0027	-	0	0	0	0	0
Azerbaijan Republic	0.0003	0.003	-	0	0	0	0
Iraq	0.0004	0.0031	0.0007	-	0	0	0
Pakistan	0.0003	0.0027	0.0007	0.0007	-	0	0
Turkey	0.0001	0.0028	0.0004	0.0005	0.0005	-	0
United Arab Emirates	0.0008	0.0035	0.0011	0.0012	0.0011	0.0009	-

## Discussion

Iran’s population is diverse in terms of ethnicity, language, and religion, and intermarriage is more common in some areas [25]. Geographical barriers, such as the Kavir Desert in the center, the Lut Desert lying in the east of the Iranian Plateau, and the Alborz and Zagros Mountains, contribute to obstacles to gene flow through the land of the Persians to other parts of the world, making Iranians unique [10]. Y-STR markers have been characterized in different regions of Iran, including Tehran [26, 27], Mazandaran and Gilan [12], Golestan [28], Isfahan [14, 26], and eastern provinces such as Razavi Khorasan, South Khorasan, and Sistan and Baluchestan [11]. Given the unique geographical composition and diversity of the Iranian population, Y-STR markers in a large sample size among Iranians have not been widely studied. Such studies have a significant effect on enriching information for use on the local and global scales. This is the first study conducted on a large Iranian sample and provides information on the genetic polymorphisms of 17 Y-STR alleles.

The results demonstrated that the value of genetic diversity ranged from 0.340 (DYS391 in Tabriz) to 0.873 (DYS385a in Mazandaran). The highest polymorphic content in the Iranian population was observed in DYS385a, whereas the lowest polymorphic STRP marker was found in DYS391. In addition, the mean gene diversity in the Iranians was 0.685, with a similar distribution to previously studied populations attributed to different regions of Iran [11, 12, 28].

The average gene diversity (GD) obtained from neighboring populations was similar to that of the Iranian population, such as Eastern Turkey [29], Turkish Cypriots [30], and Pakistan [31], possibly due to the geographical proximity of the country of residence to Iran (as a possible result of the continuous admixture of these populations). Furthermore, this suggests that the original Turkish population probably has a common ancestor with the ethnic groups living in Iran, and that their language differences do not seem to be related to genetic distance. The differences between the current and previous studies might be attributed to the smaller sample size or the use of fewer markers in the Y-STR ratio in the aforementioned studies.

The haplotype diversity was 1.00000, but the values of haplotype diversity in various areas of Iran (Tehran, Gilan, Mazandaran, Golestan, Isfahan, and eastern provinces) were 0.997, 0.9998, 0.9993, 0.9962, 0.997, and 0.9999, respectively. People from particular geographical regions shared a common haplotype; in other words, people from different regions of Iran have been separated from each other only by geographical boundaries, while they are genetically similar. Moreover, the haplotype diversity values from neighboring populations were 0.9850 in Afghanistan [32], 0.9992 in Turkish Cypriots [30], 0.99885 in the United Arab Emirates [33], 0.9986 in Eastern Turkey [29], 0.999677 in the Sindhi population of Pakistan [34], 0.9305 in Punjab Pakistan [35], 0.892 in Iraq [36], 0.9932 in Tunis [37], 0.9963 in Germany [38], 0.9941 in Italy [38], 0.9968 in China [38], 0.996 in India [38], and 1.0 in Qatar [39]. As expected, maximum haplotype diversity was observed, given the ethnic diversity of Iran’s population. In forensic medicine, high variability is the most important characteristic, and higher haplotype diversity shows better discriminating power for Y-STR haplotypes in forensic medicine. The lower HD values obtained for these countries demonstrated that the migration rate declined in the male Iranian population. On the other hand, men living in Iran were isolated, but lower HD values may also be due to the fact that the 17 Y-STRs were identified in different populations; thus, they were not very variable in the analyzed populations. The discrimination capacity (DC) value obtained for the entire country (DC = 0.997) was higher than that observed for the Iranian provinces, including Tehran (DC = 0.9) [26], Isfahan (DC = 0.938 and DC = 0.95) [14, 26], Golestan (DC = 0.8207) [28], and eastern provinces (DC = 0.9884) [11]. The DC value obtained for the entire country was higher than that for other provinces. The DC value difference between the study population and other regions of Iran might be attributed to the small sample size assessed in previous studies.

Comparing the DC value in the Iranian population with that of the neighboring populations, i.e. Afghanistan (DC = 0.6277) [32], Turkish Cypriots (DC = 0.9051 ), Eastern Turkey (DC = 0.9343) [29], United Arab Emirates (DC = 0.9003) [33], Punjab Pakistan (DC = 0.6438) [35], Tunis (DC = 0.7714) [37], and India (DC = 0.8497) [40], demonstrated that the Iranian population consisted of a relatively large number of unique haplotypes; however, these markers may not discriminate in a proper way in the neighboring populations.

Population pairwise comparisons for analyzing different regions of Iran using haplotypes in this study and Iranian haplotypes registered in the YHRD database showed an insignificant difference between them. There exists a close genetic distance in most regions of Iran with various social and geographical conditions, except for Masal and Izeh, indicating a high genetic distance from other regions of Iran. We compared the Iranian population with data obtained from the neighboring populations that are accessible in the YHRD database. Statistical analyses revealed the following: Afghanistan (FST = 0. 0027, *P* = 0.00), Pakistan (FST = 0. 0003, *P* = 0.00), Turkey (FST = 0. 0001, *P* = 0.00), Azerbaijan Republic (FST = 0. 0003, *P* = 0.00), Iraq (FST = 0. 0004, *P* = 0.00), United Arab Emirates (FST = 0. 0008, *P* = 0.00), Bahrain (FST = 0. 0014, *P* = 0.00), Egypt (FST = 0. 0008; *P* = 0.00), Kazakhstan (FST = 0. 0037, *P* = 0.00), Kuwait (FST = 0. 0018, *P* = 0.00), Lebanon (FST = 0. 0014, *P* = 0.00), Mongolia (FST = 0. 0031, *P* = 0.00), the Russian Federation (FST = 0. 0003, *P* = 0.00), Qatar (FST = 0. 0015, *P* = 0.00), and Yemen (FST = 0. 0007, *P* = 0.00).

A comparison of the Iranian population with neighboring populations demonstrated significant genetic differences, which might be due to geographical and ethnic variations. In addition, these markers were capable of male identity among the Iranian population. The present AMOVA analysis of the different regions of Iran for 17 Y-STR haplotypes compared with the data obtained from some neighboring populations (Supplementary Table 4) indicated that Turkey, Pakistan, and Iraq showed small genetic discrimination and evidence of a common ancestral lineage between populations living in these close regions. Furthermore, a multidimensional scaling (MDS) plot based on pairwise FST values between the study population and neighboring populations in the YHRD database revealed the nearest genetic distance observed between Turkey and Iran that were geographically close to each other. These populations are similar in terms of genetic diversity and are not far apart from each other.

The Y-STR haplotypes in the Iranian population were highly similar. Given this genetic similarity, it was not possible to draw a multidimensional scaling (MDS) plot between different units of the Iranian population. For some Iranian regions and cultures, intermarriage is customary; this was a limitation for us in taking samples from unrelated Iranian males to evaluate the Y chromosome haplotype.

## Conclusion

This was the first study to be conducted on a large representative of the Iranian population. The largest possible population living in different geographical regions of Iran was evaluated based on data obtained using the Yfiler Kit, which is widely used in forensic laboratories in Iran and other countries. Reliable results were obtained that could be applicable to forensic analyses and population genetics studies. Further genetic research studies on Y-SNPs could shed more light on the settlement, migration, and population admixtures of the ancient Iranian population.

### Electronic supplementary material

Below is the link to the electronic supplementary material.

Supplementary Material 1

Supplementary Material 2

Supplementary Material 3

Supplementary Material 4

## Data Availability

The data presented in this study have been deposited with links to BioProject accession number SUB12878983 in the NCBI BioProject database (https://www.ncbi.nlm.nih.gov/bioproject/).16 distinct accession numbers from Haplotype data has been uploaded to the YHRD of YA004580, YA004228, YA004259, YA004258, YA004257, YA004586, YA004587, YA004584, YA004585, YA004583, YA004582, YA004229, YA004581 for Tehran, Isfahan, Golestan, Mazandaran, Gilan, Mashhad, Birjand, Sistan and Baluchestan, Tabriz, Urumieh, Kermanshah, Kurdistan, Ilam, Hamedan, Shiraz, Ahvaz, respectively). Supplementary data associated with this article can also be found in the supplementary materials.

## Declarations

### Quality control

Quality evaluation for Y chromosome STR typing was confirmed by the correct typing of 17 Y-STR markers among five blind DNA cases presented in the YHRD database in 2012 (YC000268) (22).
